# Full-length transcriptome and RNA-Seq analyses reveal the resistance mechanism of sesame in response to *Corynespora cassiicola*

**DOI:** 10.1186/s12870-024-04728-y

**Published:** 2024-01-23

**Authors:** Min Jia, Yunxia Ni, Hui Zhao, Xintao Liu, Wenqing Yan, Xinbei Zhao, Jing Wang, Bipo He, Hongyan Liu

**Affiliations:** 1 Key Laboratory of IPM of Pests on Crop (Southern North China), Ministry of Agriculture, Key Laboratory of Crop Pest Control of Henan, Institute of Plant Protection, Henan Academy of Agricultural Sciences, Zhengzhou, Henan 450002 China; 2 Key Laboratory of Specific Oilseed Crops Genomics of Henan Province, Henan Sesame Research Center, Henan Academy of Agricultural Sciences, Zhengzhou, Henan 450002 China

**Keywords:** Sesame, full-length transcriptome, WGCNA, resistant, susceptible, *Corynespora cassiicola*

## Abstract

**Background:**

*Corynespora* leaf spot is a common leaf disease occurring in sesame, and the disease causes leaf yellowing and even shedding, which affects the growth quality of sesame. At present, the mechanism of sesame resistance to this disease is still unclear. Understanding the resistance mechanism of sesame to *Corynespora* leaf spot is highly important for the control of infection. In this study, the leaves of the sesame resistant variety (R) and the sesame susceptible variety (S) were collected at 0–48 hpi for transcriptome sequencing, and used a combined third-generation long-read and next-generation short-read technology approach to identify some key genes and main pathways related to resistance.

**Results:**

The gene expression levels of the two sesame varieties were significantly different at 0, 6, 12, 24, 36 and 48 hpi, indicating that the up-regulation of differentially expressed genes in the R might enhanced the resistance. Moreover, combined with the phenotypic observations of sesame leaves inoculated at different time points, we found that 12 hpi was the key time point leading to the resistance difference between the two sesame varieties at the molecular level. The WGCNA identified two modules significantly associated with disease resistance, and screened out 10 key genes that were highly expressed in R but low expressed in S, which belonged to transcription factors (WRKY, AP2/ERF-ERF, and NAC types) and protein kinases (RLK-Pelle_DLSV, RLK-Pelle_SD-2b, and RLK-Pelle_WAK types). These genes could be the key response factors in the response of sesame to infection by *Corynespora cassiicola*. GO and KEGG enrichment analysis showed that specific modules could be enriched, which manifested as enrichment in biologically important pathways, such as plant signalling hormone transduction, plant-pathogen interaction, carbon metabolism, phenylpropanoid biosynthesis, glutathione metabolism, MAPK and other stress-related pathways.

**Conclusions:**

This study provides an important resource of genes contributing to disease resistance and will deepen our understanding of the regulation of disease resistance, paving the way for further molecular breeding of sesame.

**Supplementary Information:**

The online version contains supplementary material available at 10.1186/s12870-024-04728-y.

## Background

Sesame (*Sesamum indicum* L.) is an important oil plant species with nutritious, medicinal, and edible uses [1]. One study suggested that globally, the total annual consumption of sesame as food and oil accounts for about 35% and 65%, respectively [2]. However, due to the impact of many biological and abiotic stresses, the sesame planting area and total production have decreased globally. *Corynespora* leaf spot (CLS) is also a serious disease of sesame in the main producing areas of China, and its pathogen *Corynespora cassiicola* can infect flowers, fruits and roots, causing leaf drying and falling off, affecting plant photosynthesis, resulting in seed wilt and reduced oil content. In addition, the remaining spores on the withered and shed sesame leaves are buried in the soil, and when sesame seeds are sown the next year, the spores begin to spread with rain and wind, forming a cycle of infection [3, 4]. For CLS, chemical or physical controls have a key role in control. However, these measures are not a permanent solution in preventing fungal infection damage. Breeding resistant sesame cultivars is regarded as the most cost-effective measure for controlling the damage caused by *C. cassiicola*. Exploring resistance genes and gene regulatory networks is a prerequisite for the molecular breeding of sesame resistance to *C. cassiicola*. Therefore, there is an urgent need to study the disease resistance of sesame to help improve the potential of sesame production.

In natural, plants are vulnerable to a variety of pathogens. Therefore, plants have evolved a complex but sophisticated and efficient immune system to fight off infection by pathogens. The application of bioinformatics technology has made new progress in our understanding of plant‒pathogen interactions [5]. Comparative transcriptional analysis using RNA-seq is a commonly used method to search for genes differentially expressed between two samples. This method is also generally used to mine plants for genes involved in disease resistance, as has been done in peanut [6], grape [7], pear [8] and apple [9]. Genomic analysis has been also used in sesame [10]. Komivi Dossa reported the RNA-seq profiles of two contrasting sesame genotypes under waterlogging stress and after recovery in 2019 [11]. Su et al. [12] identified 6736 DEGs from a transcriptome analysis and found that highly expressed genes are involved in plant hormone signal transduction and heat shock protein regulation, thereby enhancing the heat tolerance of sesame. In addition to being used to study responses to abiotic stress, the transcriptome is also widely used in biological stress studies. Radadiya et al. [13] selected a resistant sesame variety and a susceptible sesame variety and inoculated both with *Macrophomina phaseolina* at the same time; transcriptome analysis was carried out, and 1153 and 1226 differentially expressed genes, respectively, were identified. Through transcriptome analysis, relevant studies have suggested that the defence response of sesame to *Macrophomina phaseolina* is a complex biological process involving many plant hormones and disease-resistance related genes, such as those involved in JA/ET and SA signalling pathways [14]. Although RNA-seq has been used successfully for identifying *C. cassiicola*–host interactions in several different hosts, no study has yet been conducted to understand *C. cassiicola*–host interactions in sesame [15–17]. Transcriptome analysis plays an important role in screening resistance genes and identifying plant‒pathogen interactions. In addition, pathogen stress, which occurs in the reproductive stage, has the most negative effect on crop production. Therefore, it is necessary to analyse the interactions between resistant and susceptible sesame varieties and *C. cassiicola* via RNA-seq.

To the best of our knowledge, there is no information available about transcriptome differences between resistant varieties and susceptible varieties of sesame under *C. cassiicola* stress. Nevertheless, transcriptome research is one of the necessary tools for understanding biological processes. On the basis of the second generation of high-throughput sequencing platforms, RNA-seq technology cannot be used to obtain complete transcripts or assemblies accurately, and unrecognized isoforms, transcription of a homologous genes, gene expression, and super families, make it difficult to understand the meaning of biological activities at a deeper level. Full-length transcriptome sequencing based on single-molecule real-time sequencing (SMRT-seq) technology does not interrupt the RNA fragment and instead involves the direct reverse transcription of the obtained full-length cDNA. The ultra-long read (median10 kb) system of the platform houses the sequence information of a single complete transcript, which does not need to be assembled for later analysis [18–20].

The acquisition of full-length cDNA sequences is the basis of the most important structural and functional genomics research. Full-length transcriptome contain expression information of gene sequences, which is important for functional analysis at the transcription and translation levels. In the past few years, an increasing number of PacBio full-length transcriptome have been sequenced and assembled. These studies have helped us to identify numerous new genes and alternatively spliced isoforms in many species, including potato [21], sorghum [22], *Larix kaempferi *[23], Italian ryegrass root [24] and kiwifruit [25]. Overall, the above studies demonstrated that third-generation sequencing complements second-generation sequencing in the quantitative determination of eukaryotic transcripts and contributes to the discovery of an increasing number of alternatively spliced isoforms [26]. In this study, transcript-level responses of resistant and susceptible varieties of sesame infected by *C. cassiicola* were explored via second- and third-generation sequencing technologies. Our results provide bases for a better understanding of the resistance response of sesame and potential candidate genes for further sesame resistance studies.

## Results

### Statistical analysis of transcriptome sequencing data of resistant and susceptible sesame varieties

To further improve the accuracy of the PacBio SMRT-seq results, thirty-six Illumina RNA-seq libraries constructed from sesame leaves treated at different time points before and after inoculation were subjected to second-generation sequencing to correct the polished isoforms of PacBio SMRT-seq with Lordec software [27] and to quantify the full-length transcripts that had been obtained. According to the conditions full passes >  = 3 and sequence accuracy greater than 0.9, a circular consensus (CCS) sequence was extracted from the original sequence and corrected (Table S1).

**Fig.1:**
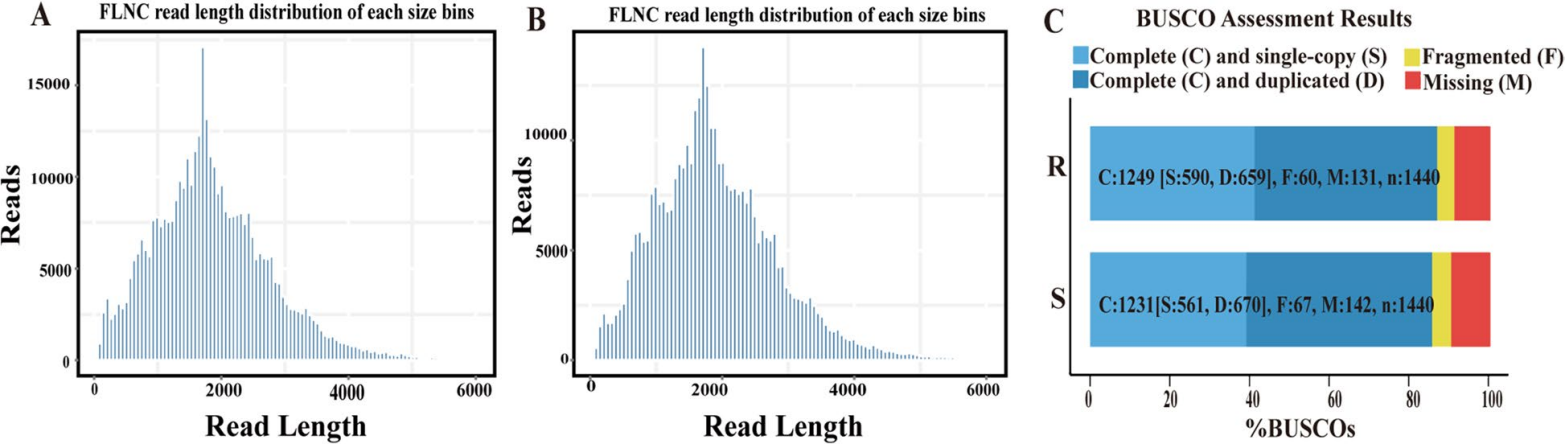
Summary of PacBio RS II single molecule real-time (SMRT) sequencing. **A** FLNC read length distribution of each size bin of R; **B** FLNC read length distribution of each size bin of S; **C** Transcriptome integrity assessment results of R and S (Note: n and the corresponding number in the figure are the single-copy gene set of related species and the number of genes in the gene set)

Full-length transcriptome sequencing of resistant and susceptible sesame were completed, and 35.75 Gb and 33.07 Gb of data, respectively, were obtained. SMRT-seq yielded 492,210 and 454,360 CCS reads after polishing, among which 388,993 and 367,963 full-length nonchimeric (FLNC) reads were obtained (Fig. 1A-B). In total, 135,433 and 126,193 consensus sequences were obtained by clustering the FLNC sequences, and 135,357 and 126,102 high-quality consensus sequences were obtained (Tables S2 and S3). The integrity of the high-quality full-length transcripts was evaluated by BUSCO. There were 2880 complete BUSCOs in both the R and S groups, with 1249 complete BUSCOs (43.37%) in the R group and 1231 complete BUSCOs (42.74%) in the S group (including single-copy and duplicated BUSCOs), respectively (Fig. 1C).

### Analysis of alternative splicing (AS)

Precursor mRNAs (pre-mRNAs) generated by gene transcription can be spliced in a variety of ways. Different exons are selected to produce different mature mRNAs, which can be translated into different proteins and constitute the diversity of biological traits. This post-transcriptional mRNA processing is called variable splicing or alternative splicing. The generation of fusion transcripts may be related to variable splicing, and full-length transcriptome sequencing can be used to identify the structure of fusion transcripts accurately.

**Fig. 2:**
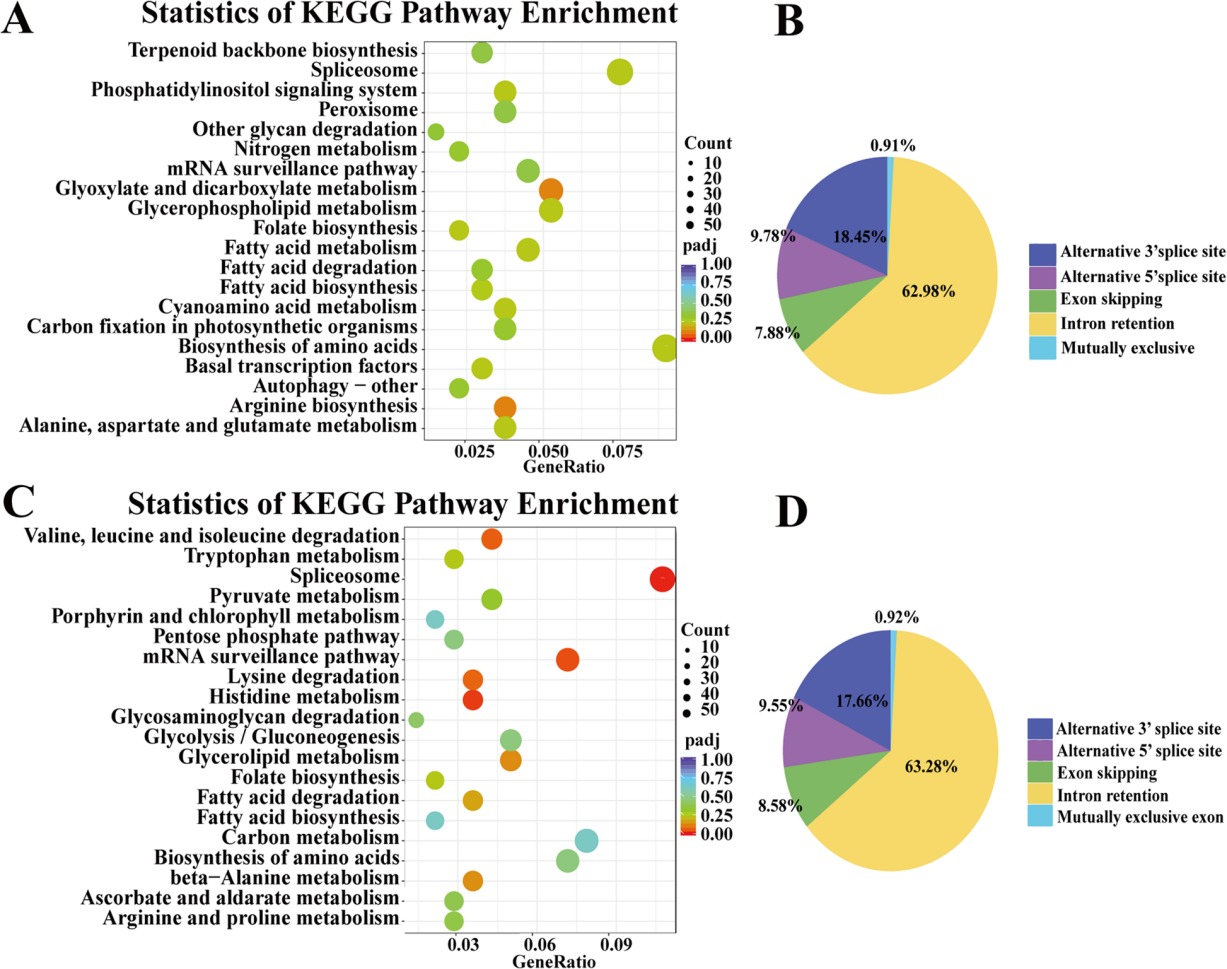
Identifcation of alternative splicing (AS) events and functional analysis. **A** KEGG enrichment analysis for differential AS genes in R; **B** Statistics of the number of AS events in R; **C** KEGG enrichment analysis for differential AS genes in S; **D** Statistics of the number of AS events in S

We counted the number of the above five variable splicing events detected in the transcripts (Fig. 2). In R and S, 29,082 and 28,799 AS events were predicted, respectively. We found that intron retention constituted the highest proportion– 62.98% and 63.28%, respectively, but mutually exclusive exons constituted the lowest proportion – only 0.91% and 0.92%, respectively (Fig. 2B, D). In the KEGG pathway enrichment, the differential AS genes of R were significantly enriched in “Biosynthesis of amino acids”, “Spliceosome”, “Glyoxylate and dicarboxylate metabolism” and “Glycerophospholipid metabolism”; interestingly, the differential AS genes of S were significantly enriched in “Spliceosome”, “Carbon metabolism”, “mRNA surveillance pathway” and “Biosynthesis of amino acids” (Fig. 2A, C). All the differential AS genes sets were enriched in the spliceosome. Glyoxylate and dicarboxylate metabolism was found to be a unique pathway in R, while Carbon metabolism was unique to S.

### Functional notes and structure analysis of transcripts

**Fig. 3:**
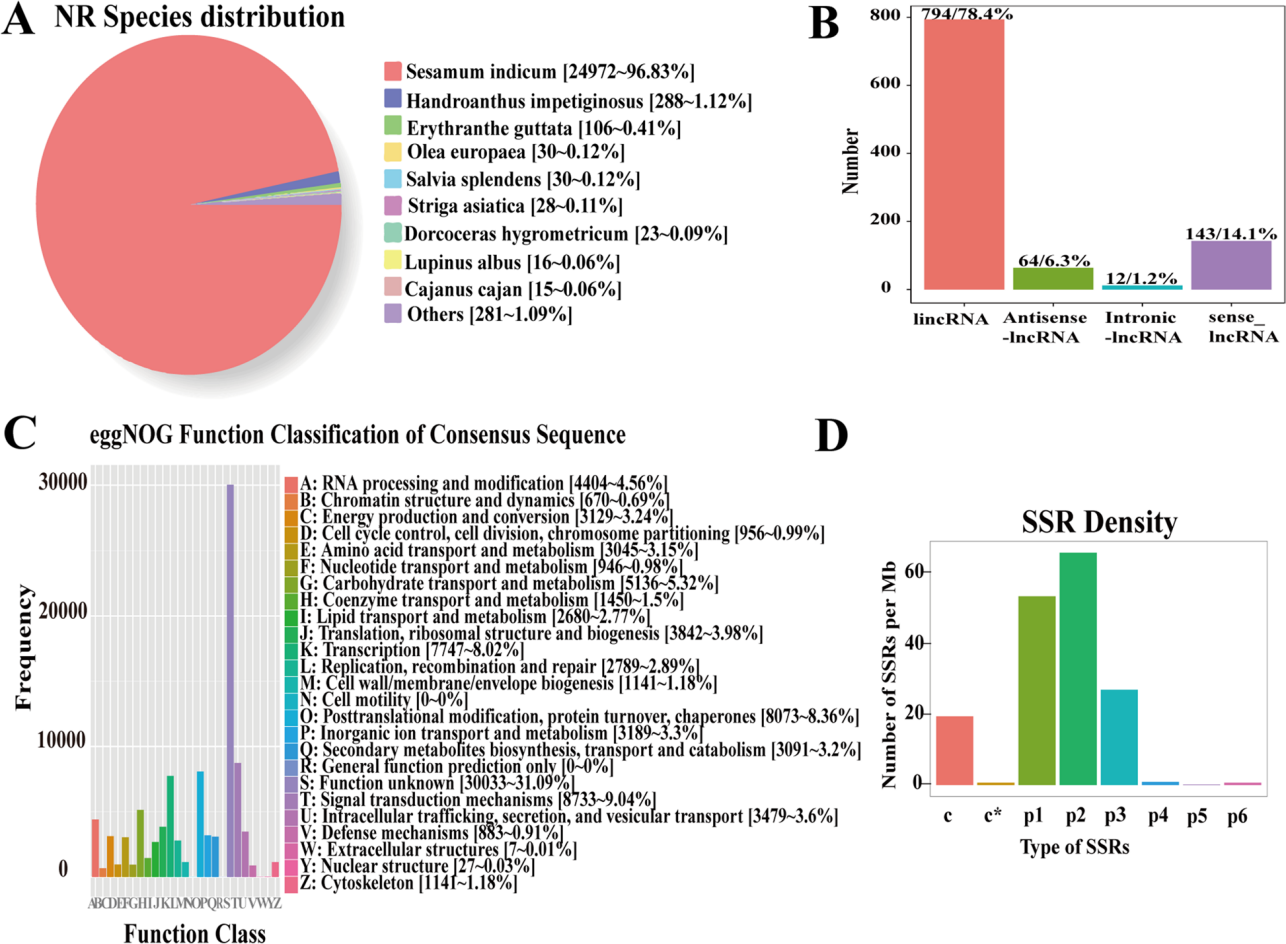
Functional notes and structure analysis of transcripts. **A** Comparison of homologous sequences among species in the NR database; **B** lncRNA position classification drawing. Note: Four different types of lncRNAs (lincRNA: long non-coding RNAs in intergene regions; Antisense- lncRNA: Antisense long non-coding RNA; lntronic-lncRNA: intron long non-coding RNA; sense_lncRNA: positive-sense long non-coding RNA), and the ordinate is the corresponding lncRNA number; **C** EggNOG function classification of consensus sequence; **D** Classification and repetition numbers of simple repeat sequences (SSRs). Mononucleotides: (p1), Dinucleotides: (p2), Trinucleotides: (p3), Tetranucleotides: (p4), Pentanucleotides: (p5), Hexanucleotides: (p6), compound SSRs: (c) and compound SSRs with overlapping positions: (c*)

The new transcripts obtained from the variable splicing analysis were functionally annotated, and the number of transcripts annotated by each database was counted (Table S4). NR sequence alignment was used to predict the species most closely related to sesame. Through sequence alignment, 95.14% of the sequences were consistent with the published sesame transcriptome sequence (Fig. 3A). A total of 74,877 transcripts were annotated; of these, 65,738 transcripts were annotated in the eggNOG database (Fig. 3C). The lncRNAs were classified and mapped according to their positions in the reference genome annotation information (Fig. 3B). The density distribution of different SSR types was statistically analysed. The most predominant SSR type was the dinucleotide repetition type, followed by the Mononucleotide repetition type (Fig. 3D).

### Analysis of differentially expressed genes in response to *C. cassiicola*

**Fig. 4:**
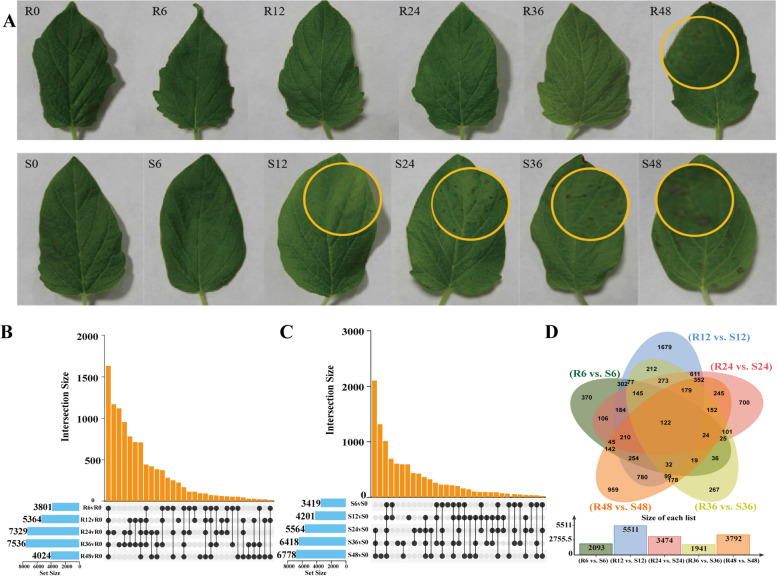
The variation of DEGs in R and S. **A** Leaf symptoms in the R group and S group after inoculation with *C. cassiicola*; **B** Comparison of differentially expressed genes in the R group at different time points; **C** Comparison of differentially expressed genes in the S group at different time points. **D** Comparison of differentially expressed genes in the R group and S group at different time points

To observe the leaf symptoms of resistant and susceptible varieties after *C. cassiicola* infection, we inoculated sesame leaves with *C. cassiicola*, and sterile water was used to serve as a mock inoculation. The leaves were observed at 0 (preinoculation), 6, 12, 24, 36, and 48 h post-inoculation (hpi). Spot symptoms developed at 12 hpi, and mild spots were observed at the inoculation site in the S group. In addition, before mild spots were observed in the R group, the S group were infected and significant punctate spots appeared at 24 hpi. The R group developed slight symptoms resulting from inoculation at 48hpi (Fig. 4A); Samples were collected at 0 (preinoculation), 6, 12, 24, 36 and 48 hpi for transcriptome sequencing, and each time point contained three replicates.

The number of differentially expressed gene sets was counted in Table S5. In the S group, the number of DEGs showed a gradually increasing trend from 0 to 48 hpi; in the R group, the number of DEGs increased gradually from 0 to 36 hpi, but decreased at 48 hpi (Fig. 4B, C). It is speculated that this change trend may be related to the function of disease resistance. As a result, a total of 5,364 (2,436 up-regulated and 2,928 down-regulated) genes were identified as potential resistance responsive genes in R at 12 hpi. Similarly, a total of 4,201 (2,136 up-regulated and 2,065 down-regulated) genes were found to be associated with positive and negative responses to *C. cassiicola* in S at 12 hpi (Fig. 4D).

As shown in Fig. 4D, by comparing the variation in DEGs between the R and S group at the same time point, we found that the number of DEGs between R and S was the highest at 12 hpi. Combined with the phenotypic observations of sesame leaves inoculated at different time points, these results indicated that both R and S were subjected to *C. cassiicola* for 12 hpi, which was the key time point that led to the resistance difference between the two varieties at the molecular level.

### Analysis of transcriptome data at different time points between the R and S group under *C. cassiicola* stress

PCA (Fig. S1) showed that S0, S6, and S12 and R0, R6, and R48 were grouped together (left), while S24, S36, and S48 and R12, R24, and R36 were clearly clustered together (right). These results indicated that a higher similarity in transcriptional programming and obvious transcriptional differences between R and S occurred under *C. cassiicola* stress for 0–12 hpi and under *C. cassiicola* stress for 24–48 hpi, respectively. Furthermore, the difference of R and S gene expression at 12 hpi also indicated that 12 hpi was the key time point leading to the resistance difference between the two varieties at the molecular level.

**Fig. 5:**
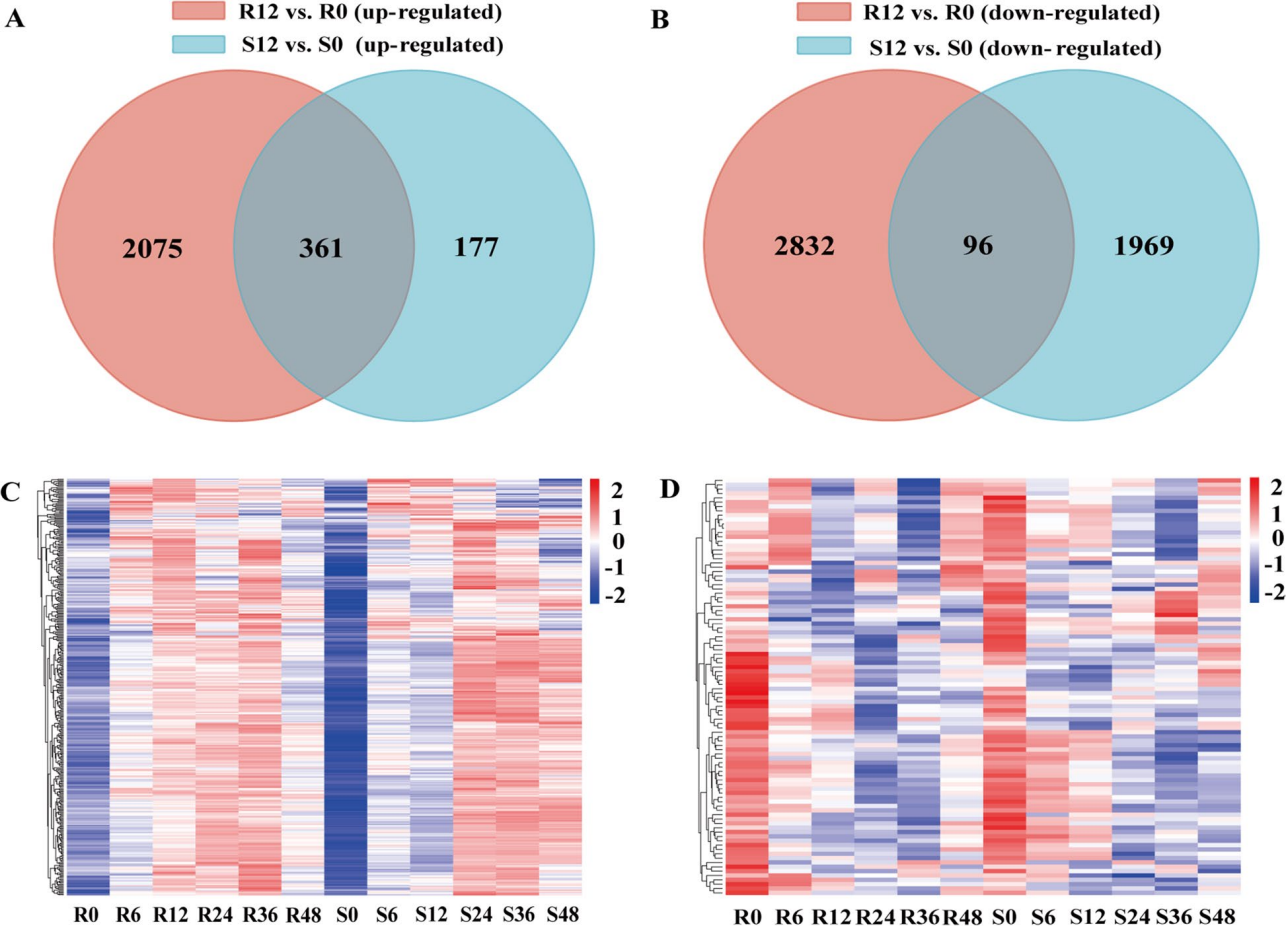
Heatmap of differentially expressed resistance genes between R and S. **A** Venn diagram comparing up-regulated DEGs in R and S at 12 hpi; **B** Venn diagram comparing down-regulated DEGs in R and S at 12 hpi; **C** Heatmap of up-regulated common genes in R and S at 12 hpi; **D** Heatmap of down-regulated common genes in R and S at 12 hpi

R had more up-regulated and down-regulated genes than S did after inoculation at 12 hpi. These genes were identified as potential contributors to the R group having higher resistance ability than the S group. In addition, among these potential resistant responsive genes, only 361 up-regulated genes overlapped in R and S (Fig. 5A, B). These 361 up-regulated genes are also potential positive responsive genes in R. Therefore, we believe that these 361 up-regulated genes are resistance genes that lead to the higher resistance ability of R. At 6 hpi, there was no significant difference in up-regulated gene expression between R and S, but more than half of these genes had higher expression in the R group than in the S group at 12 hpi (Fig. 5C), this result indicated that the key time point for R to initiate resistant defense response was 12 hpi. Interestingly, most of the genes in S were down-regulated at 12 hpi and up-regulated at 24 hpi compared with those in R, indicating that the defence response genes in S after *C. cassiicola* infection were not induced successfully during the early infection stage, which might be the cause of *C. cassiicola* susceptibility in the S group. At the same time, there was no significant difference in the expression of 96 down-regulated genes in R and S, indicating that down-regulated genes were not closely related to disease resistance (Fig. 5D). On the whole, by further comparing R and S, it was found that the up-regulated of DEGs induced by pathogens in R may be the basis of resistance enhancement.

### Identification and functional annotation of differentially expressed resistant responsive genes between R and S

**Fig. 6:**
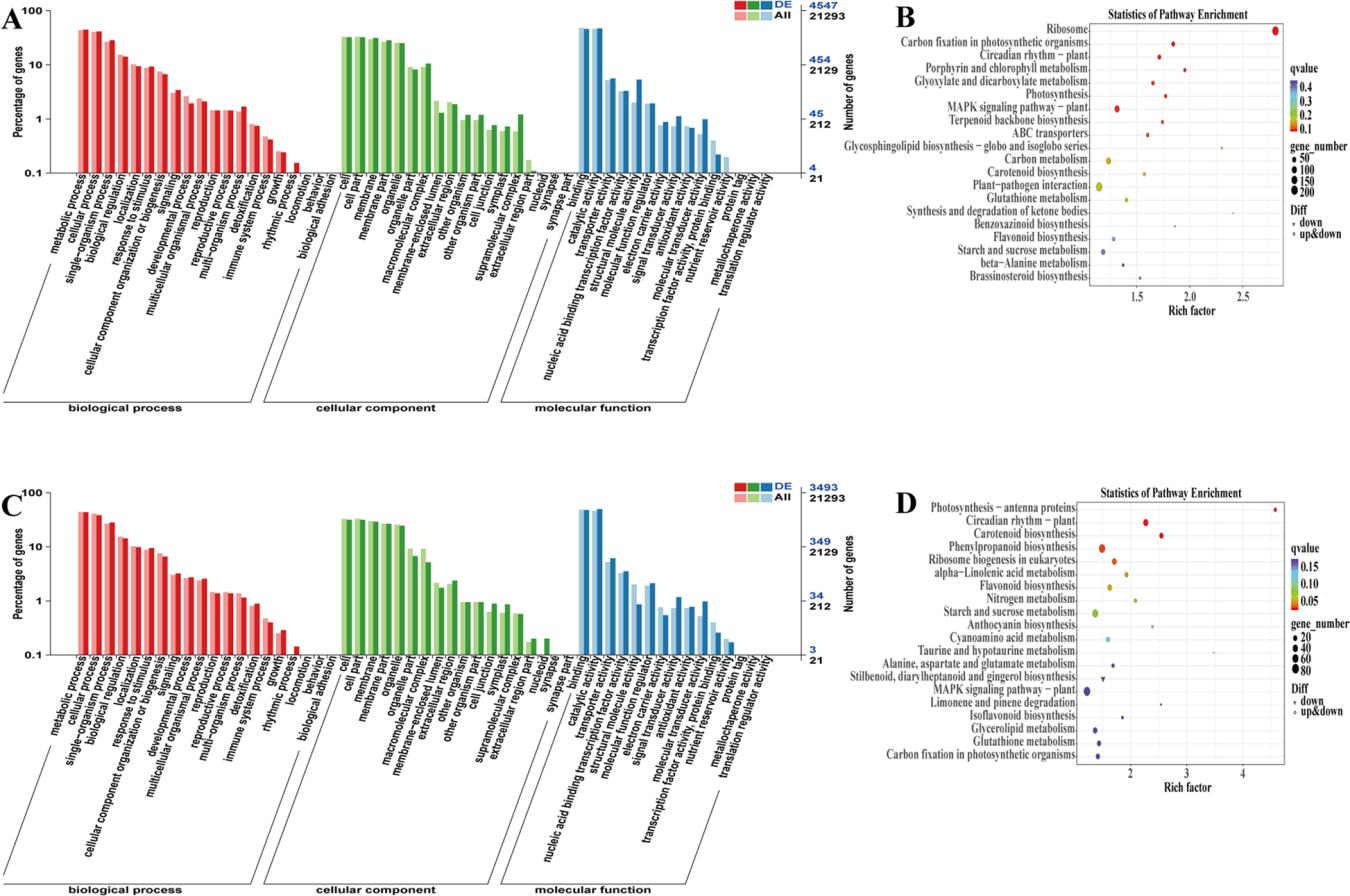
Functional annotations of DEGs in R and S under *C. cassiicola* stress. **A** GO classification of DEGs in R; **B** KEGG pathway enrichment of DEGs in R; **C** GO classification of DEGs in S; **D** KEGG pathway enrichment of DEGs in S

We conducted pairwise comparisons between uninoculated and *C. cassiicola*-inoculated leaves (R12 vs. R0, S12 vs.S0) to identify genes that respond to *C. cassiicola* infection at each time point in each variety. According to the GO annotation analysis, the DEGs were divided into the biological process, molecular function, and cellular component categories (Fig. 6A, C).

In the biological process category, the majority of DEGs belonged to metabolic process and cellular process. In the cellular component category, the number of DEGs in the cell and membrane GO terms were most common. For the molecular function category, the most abundant DEGs were annotated under binding and catalytic function.

TopGO analysis further revealed that the plant hormone signal transduction, plant–pathogen interaction and carbon metabolism were among the most highly enriched terms (Fig. S2). To identify active biological pathways enriched with DEGs in the two sesame varieties, the KEGG pathway database was searched. Based on the KEGG enrichment analysis, the top 20 top-ranking pathways were determined; these are presented in the form of a bubble diagram (Fig. 6B, D). Under the stress of *C. cassiicola*, the DEGs were enriched mainly in carbon metabolism, starch and sucrose metabolism, biosynthesis of amino acids and phenylpropanoid biosynthesis. These results indicated that there were significant differences in the transcription levels of carbon metabolism and phenylpropanoid biosynthesis genes between the different varieties under *C. cassiicola* stress.

In the R group, the MAPK signalling pathway - plant, plant–pathogen interaction, ribosome, starch and sucrose metabolism, carbon metabolism and glutathione metabolism were also enriched. In the S group, the MAPK signalling pathway-plant, phenylpropanoid biosynthesis, starch and sucrose metabolism, and circadian rhythm-plant, which may play significant role in response to *C. cassiicola*, were all enriched (*P* < 0.05). Additionally, the pathways of the MAPK signalling pathway-plant and starch and sucrose metabolism were both obviously enriched in R and S (Fig. 6B, D). These results indicated that *C. cassiicola* stress could regulate the complex biological pathways of sesame, and there were shared and different pathways between varieties that may function in response to *C. cassiicola*. Therefore, plant–pathogen interactions and plant hormone signal transduction may contribute to differences in the resistance of R and S.

### Co-expression network analysis identified key modules correlated with resistance to *C. cassiicola*

**Fig. 7:**
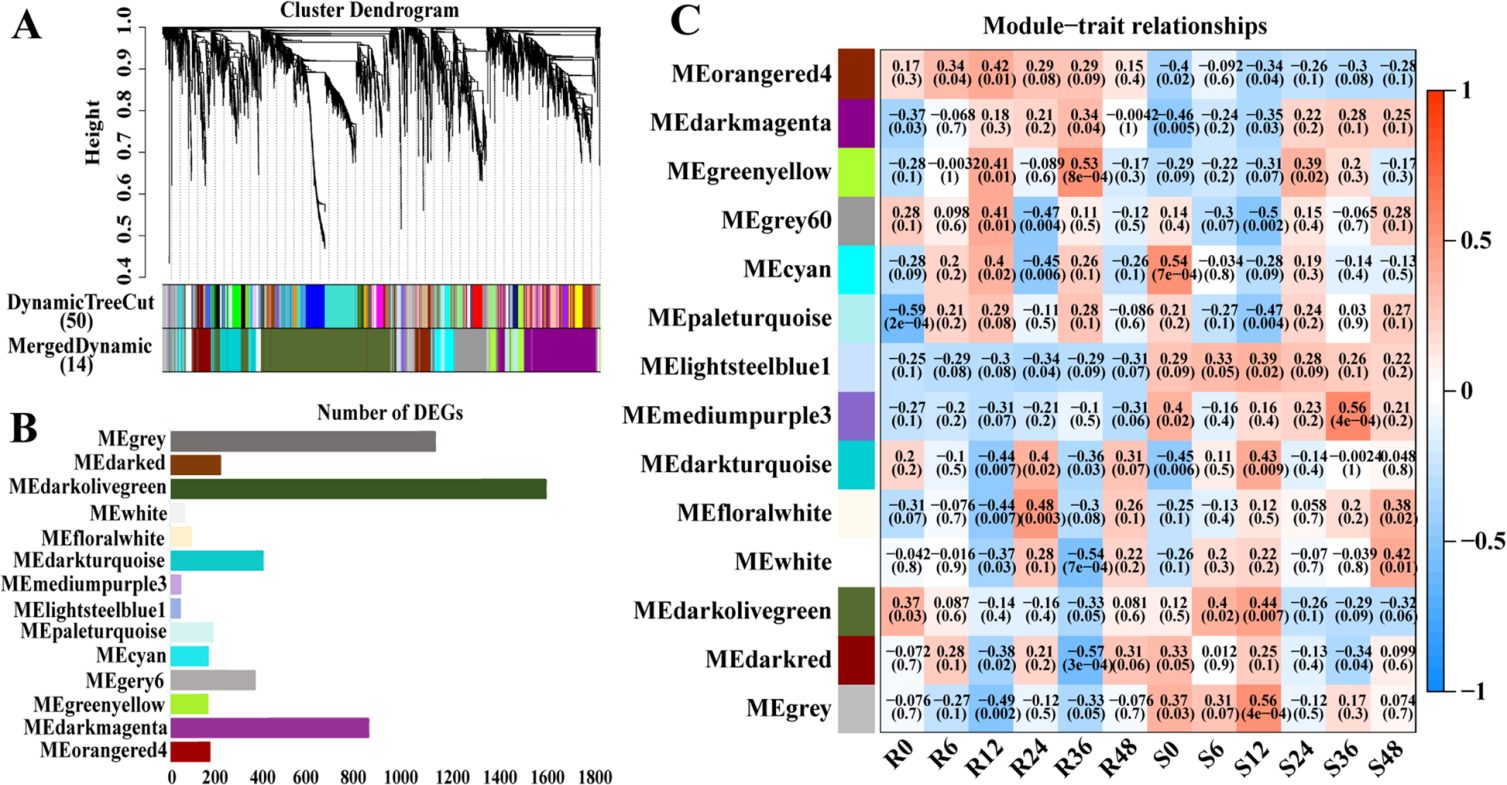
Identification of DEGs by WGCNA. **A** Module level clustering diagram; **B** Summary of the number of module genes; **C** Module-trait associations; (Each column corresponds to different time points in R and S, and each row corresponds to the characteristic gene of the module. The correlation between two is indicated in the module by the pearson correlation coefficient and *p* value in parentheses

To mine the hub genes expressed in response to *C. cassiicola*, weighted gene co-expression network analysis (WGCNA) was performed to link gene expression levels with different time-points. A total of 5551 filtered genes (with FPKM > 0.1) differentially expressed between R and S post-inoculation were further investigated by WGCNA. Each branch represented a co-expression module with different colours representing different modules. These DEGs were clustered into fourteen modules labelled with different colours (Fig. 7A, B). Furthermore, to identify the modules that were significantly associated with different time points in R and S, module–trait correlation relationships were constructed (Fig. 7C).

We found that the gene expression patterns in two modules (MEorangered4 and MEdarkmagenta) were significantly correlated with the infection time of R and S. For instance, the expression levels of genes in the MEorangered4 module showed a positive correlation in the R group, while genes in the module showed a negative correlation in the S group. In addition, the expression levels of genes in the MEdarkmagenta module were low before 12 hpi in the R group and S group, but then gradually increased at 24 hpi and 36 hpi to various extents; specifically, the MEdarkmagenta module was found to be associated with specific infection stages. WGCNA results showed that gene expression in the two aforementioned modules was specifically correlated with resistance. The expression patterns of the genes in these two modules are shown in Fig. 7C.

**Fig. 8:**
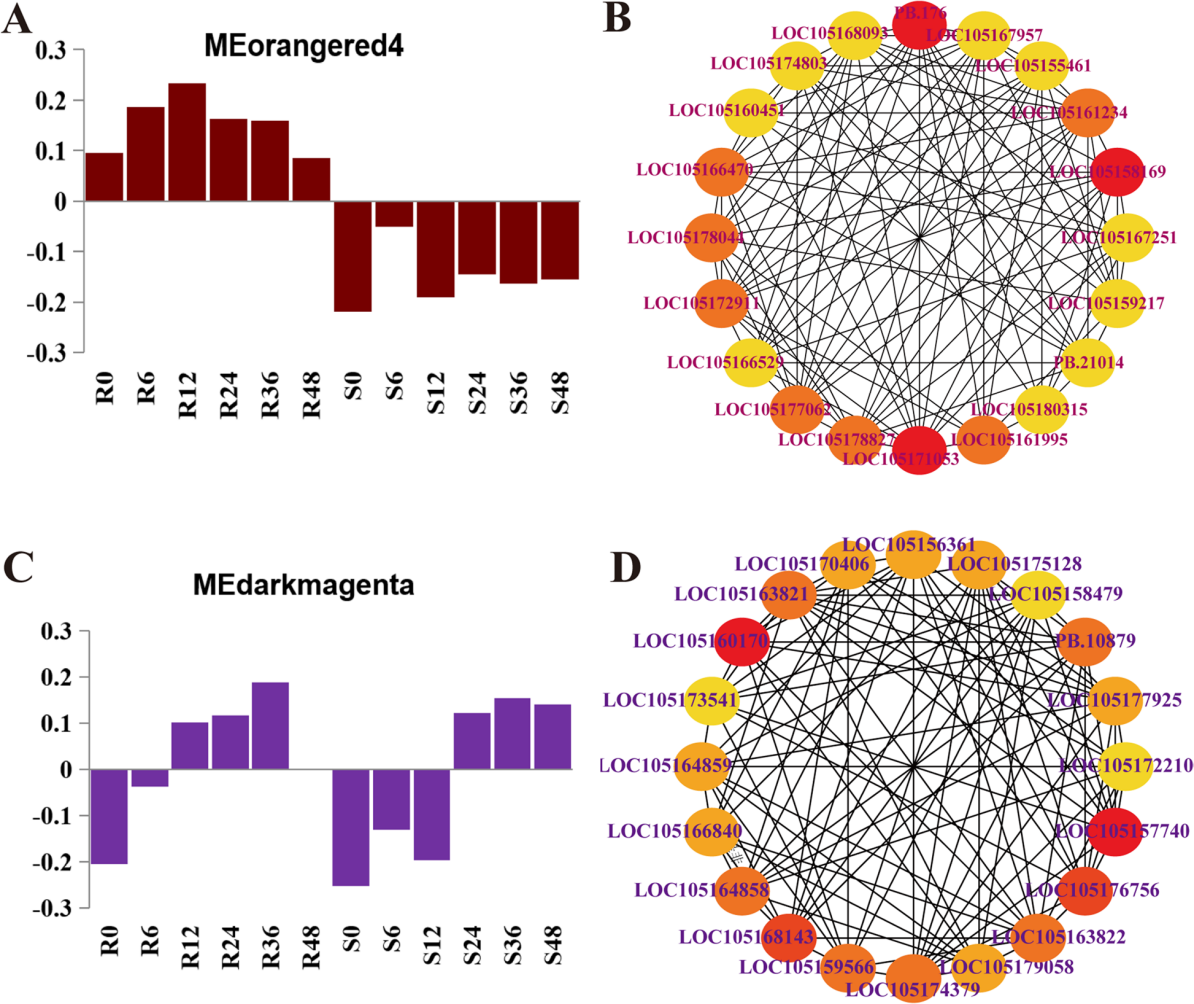
The expression patterns of the co-expressed genes in the representative modules. **A** MEorangered4 module; **B** Hub genes interaction network diagram of MEorangered4 module; **C** MEdarkmagenta module; **D** Hub genes interaction network diagram of MEdarkmagenta module

In order to mine the key genes and understand the relationship between the genes within the modules, Cytoscape software and plugin of Cyto-Hubba [28] was used to construct gene networks, which are shown in Fig. 8. An analysis was conducted on the top 20 nodes of connectivity in the MEorangered4 and MEdarkmagenta modules, and we found some hub genes related to disease resistance in the modules (Fig. 8).

In the MEorangered4 module, two resistance genes were identified, namely, LOC105177062 (glutathione S-transferase F9), LOC105172911 (chromatin remodelling protein SHL), where LOC105172911 (PHD) encodes a transcription factor.

In the MEdarkmagenta module, six hub genes, namely, gene-LOC105157740 (glutathione S-transferase), gene-LOC105174379 (glutathione S-transferase), gene-LOC105175128 (RLK-Pelle_DLSV), gene-LOC105177925 (RLK-Pelle_WAK_LRK10L-1), gene-LOC105169175 (bZIP), and gene-LOC105166840 (PLATZ), are considered to be involved in the response to various biotic or abiotic stresses, these genes encoded two protein kinases and two transcription factors. Hence, these hub genes and biological pathways might play a vital role in modulating the defence response to *C. cassiicola* infection in sesame.

### TF and PK responses to *C. cassiicola*

**Fig. 9:**
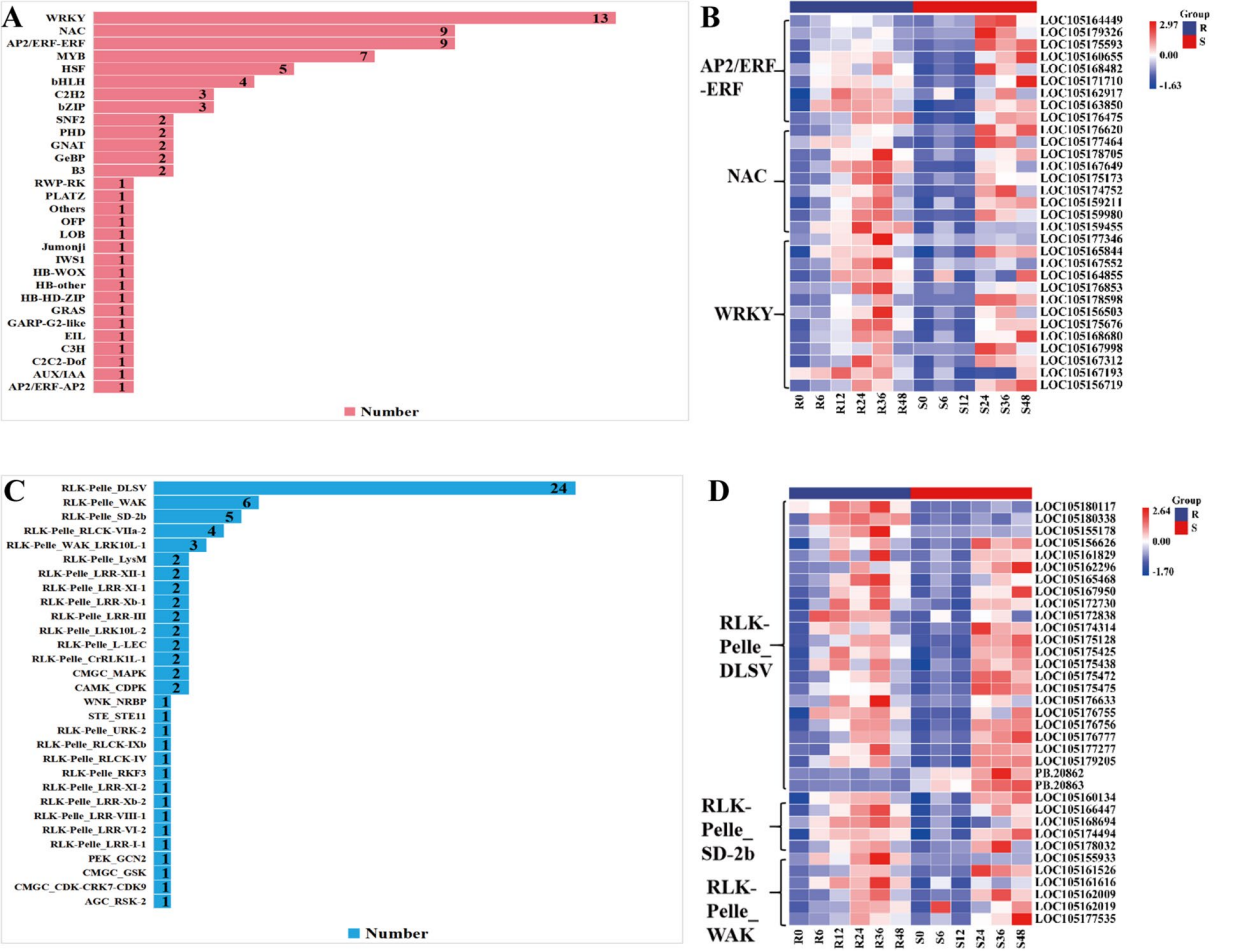
Identification of TFs and PKs. **A** The number of predicted TFs in the transcriptome data; **B** Heatmap of the differentially expressed hub DEGs encoding TFs; **C** The number of predicted PKs in the transcriptome data; **D** Heatmap of the differentially expressed hub DEGs encoding PKs

We identified 80 TFs belonging to 30 TF families in these two modules (MEorangered4 and MEdarkmagenta modules). The WRKY, AP2/ERF-ERF, NAC, MYB, HSF, bHLH, C2H2, and bZIP families of TFs were significantly enriched in R and S under *C. cassiicola* stress, of which WRKY, AP2/ERF-ERF and NAC were predominant, with 13, 9 and 9 differentially expressed TFs, respectively (Fig. 9A, B). For the WRKY, AP2/ERF-ERF and NAC families, the response of *C. cassiicola* stress was very similar. In the R group, the expression of some genes was significantly up-regulated, but in the S group, the up-regulated expression of these genes was not obvious (Fig. 9B). Moreover, the R group resulted in a much greater number of up-regulated genes in these TFs, and after inoculation with *C. cassiicola*, the responses were typically higher (in terms of fold-change) than were those of the S group.

We identified 77 PKs belonging to 30 families in these two modules, and the three most abundant PK families were RLK-Pelle_DLSV, RLK-Pelle_SD-2b, and RLK-Pelle_WAK. As shown in Fig. 9C and D, 77 PK genes were differentially expressed in these two modules, of which 35 genes were common to both varieties and showed similar expression patterns. For instance, several protein kinase genes, including RLK-Pelle_DLSV, RLK-Pelle_SD-2b, and RLK-Pelle_WAK, were up-regulated in both R and S (Fig. 9D). Our results suggest that the genes in these two modules may be involved in disease resistance during the infection process of *C. cassiicola*, but this finding requires further verification.

### Sesame resistant-related genes and pathways

In order to explore the disease resistance mechanism of sesame, we examined changes in the transcription of potential resistance-related genes in R and S. Most of the genes related to disease resistance were induced to be expressed at the early stage of infection (12 hpi). Furthermore, other DEGs were found to be enriched in the main resistance-responsive metabolic pathways such as the carbohydrate metabolism, starch and sucrose metabolism, and secondary metabolism pathways. The accumulation of starch and carbohydrates in leaves is a common phenomenon after the pathogen infects plants. In our study, it was found that the expression of genes related to the metabolic pathway of starch and sucrose changed greatly after infection by *C. cassiicola* in both R and S. These genes (LOC105174799, LOC105166206, LOC105166205, LOC105173883, LOC105173367, LOC110011237) were highly enriched in the starch and sucrose metabolism pathway, among which the gene LOC110011237 (raucaffricine-O-beta-D-glucosidase) began to be highly expressed at 6 hpi with *C. cassiicola*, and the expression trend decreased gradually at 36 hpi.

**Fig. 10:**
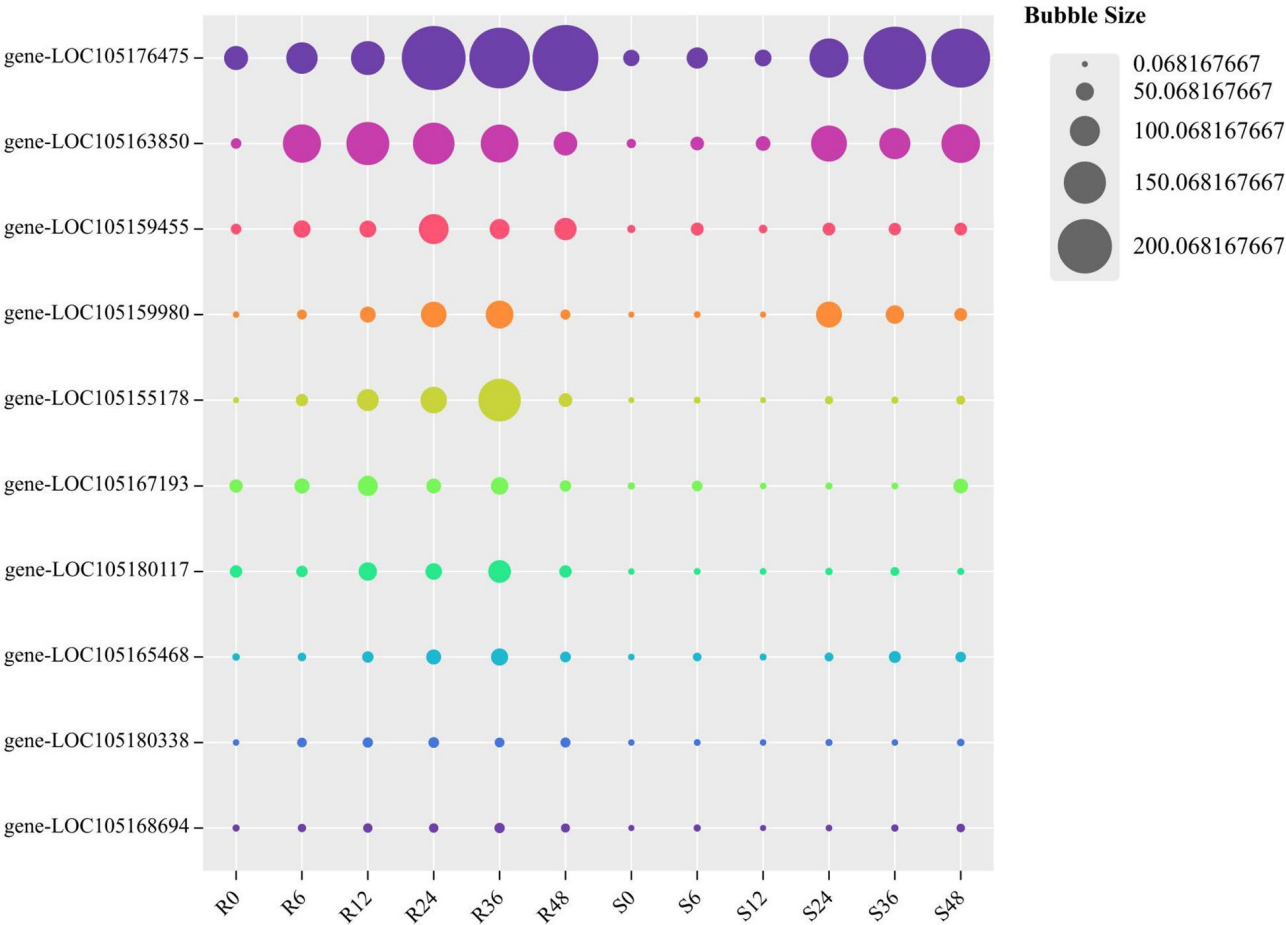
Bubble Chart showing the most significant DEGs at each time point comparison of both R and S. The DEGs at 6 time points are depicted by respective colour codes

**Fig. 11:**
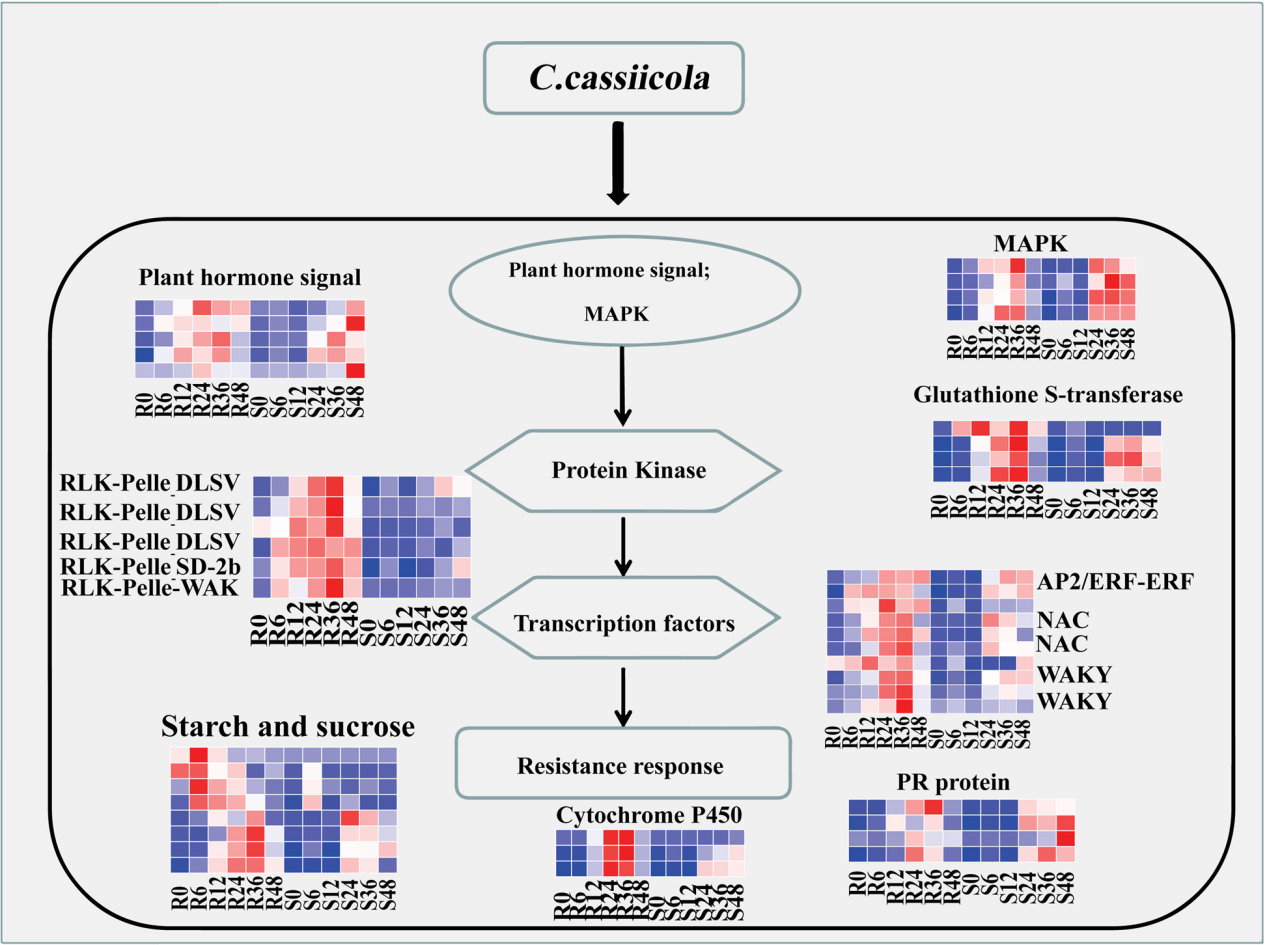
A pattern response diagram of sesame to *C. cassiicola*

Some protein kinases and transcription factors, including RLK-Pelle_DLSV, RLK-Pelle_SD-2b, RLK-Pelle_WAK, WRKY, AP2/ERF-ERF, and NAC, play vital roles in the resistance response to *C. cassiicola*. These PK- and TFs-encoded differentially expressed genes were significantly highly expressed in the R group, but were expressed at low levels in the S group (Fig. 10). The description of DEGs is provided in Table S6. These TFs and PKs that may be involved in *C. cassiicola*-mediated defence responses through the activation of disease resistance signalling and downstream defence pathways. It is suggested that these differentially expressed genes play a key role in the response to *C. cassiicola* stress in resistant variety. Based on data analysis of differential gene expression in the transcriptome, a pattern response diagram of the interactions between sesame and *C. cassiicola* was shown as Fig. 11.

### Validation of RNA-Seq by qRT‒PCR

**Fig. 12:**
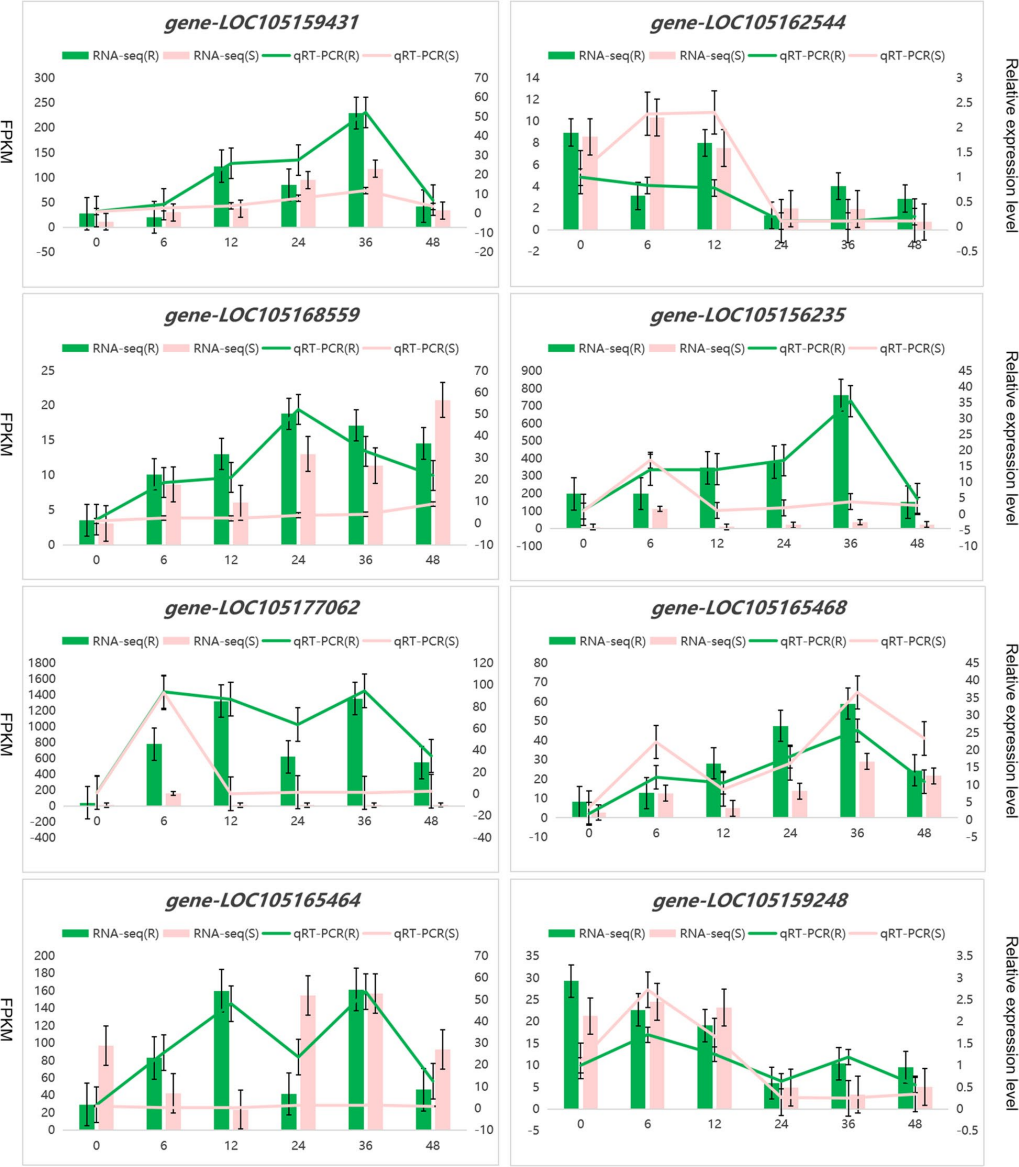
Quantitative RT‒PCR validation data of genes in R and S

To validate the reliability of the RNA-seq data, 8 DEGs were selected and tested by qRT‒PCR; these genes encoded RLK-Pelle_DLSVs (LOC105165468, LOC105165464), RLK-Pelle_LRRs (LOC105162544, LOC105168559), a kirola-like protein (LOC105156235), glutathione S-transferase (LOC105177062), and a zinc-finger homeodomain protein (LOC105159248) from the MEorangered4 and MEdarkmagenta modules. The trends of these genes at different treatment time points observed via qRT‒PCR were similar to those observed via RNA-seq, which validated the reliability of our transcriptome data (Fig. 12).

## Discussion

*C*. *cassiicola* is the causal agent of the most common leaf disease of plants in the world. This fungus can infect the flowers, stems and leaves of sesame, and *Corynespora* leaf spot is the most substantial disease. Sesame is an oilseed crop species challenged by many biotic stresses. At present, transcriptome analysis data of sesame charcoal rot, stem blight and other diseases have been reported [13]. To date, the molecular mechanisms of resistance to *Corynespora* leaf spot in sesame have not yet been reported. Due to the advantages of transcriptome analysis in the study of plant‒pathogen interactions, transcriptome analysis has been widely used to study the mechanisms of crop stress response [29–31]. Previous large-scale sequencing of cDNA has been instrumental for gene discovery in sesame, but the sequences rarely cover entire transcripts due to the limitation of NGS technologies [32]. SMRT sequencing produces longreads and is particularly useful for non-model species for which whole-genome sequencing data are lacking [33, 34]. With the continuous improvement of sequencing technology, the application of third-generation sequencing technology in the study of plant interaction is also very common [35–37]. Here, our results provide the first comprehensive, high-quality, full-length transcriptome of the sesame cultivars ‘Henan No.1’ and ‘Jinzhi No.3’ via a combination of SMRT-seq and RNA-seq, with correction of SMRT reads using Illumina reads. SSR is a series repeat sequence consisting of several nucleotides (generally 1 ~ 6) as repeating units, which is tens of nucleotides long. The sequences on both sides of each SSR are generally relatively conservative single-copy sequences. As a molecular marker, SSR is of great significance in plant disease resistance breeding [38, 39]. Maanju et al. [40] used a set of aphid specific 10 SSR markers to analyze the genetic diversity and population structure of 109 barley genotypes against *R. maidis*, and They found that only 2 genotypes were found to be resistant against Corn-leaf aphid (CLA). Sonah et al. [41] established an online database of genome-wide SSR markers called ‘BraMi’ (Brachypodium microsatellite markers), enriching resources for plant genome research. By analysing the sequence structure of the newly discovered transcripts, we predicted 50,832 SSR sequences, which provided abundant molecular markers for the later development of sesame resistance genetic breeding. In the study, the short reads produced by 36 RNA-seq libraries were used for alignment against the PacBio datasets to calculate FPKM values. Thus, all the transcripts used for subsequent analysis are complete, reducing misassembly of genes, especially in those gene families with high sequence identity. These data may greatly help researchers study sesame at the molecular level.

When plants encounter stress, AS can result in a series of defensive stress responses [21]. Recent studies have revealed that several defence genes undergo alternative splicing that is often affected by pathogen infection [42]. AS may play an important role as traditional transcription control in defence against pathogen infection [43]. Li et al. identified a spliceosome protein (SR45a) involved in the post-transcriptional regulation of the *Arabidopsis thaliana* salt tolerance [44]. By using full-length transcriptome sequencing, we also found significant differences in AS types between resistant and susceptible varieties of sesame. In this study, 29,082 and 28,799 AS events were predicted in R and S, respectively, each with different alternative splicing events. These results suggest that different alternative splicing events may be involved in the resistance. However, the underlying regulatory mechanism of how sesame regulates resistance signals through multiple transcript isoforms is still poorly understood, and further studies are needed. Transcriptome studies on *C. sublineola-*infected sorghum have shown that AS plays a crucial role in the defence response to fungal invasion [43], which is similar to the conclusion of this study.

Through differential gene pathway analysis, Su et al. [12] suggested that plant signal transduction participated in and enhanced the heat tolerance of sesame. To understand the molecular mechanisms of this oilseed crop species in response to salt stress. Zhang et al. [45] examined the transcriptome and proteome profiles of two sesame varieties with contrasting tolerances to salinity. Although plants can produce some defensive responses under the influence of the external environment, such as drought, high salt and temperature, the signal defence mechanism of pathogen infection still needs to be further explored [46–48]. Our study also showed that sucrose and starch metabolism-related genes were significantly altered after treatment. Furthermore,the most enriched genes were also associated with phytohormones. Based on these data, we proposed that plant hormone signals aiming to initiate defence regulation in response to the host are essential for the subsequent development of sesame.

Previous transcriptome analysis has revealed that starch and sucrose metabolism and plant hormone signal transduction may play important roles in the resistance mechanism of plants [49, 50]. In this study, 17 DEGs were significantly enriched in the plant hormone signal transduction pathway. Among these DEGs, those related to resistance, peroxidase activity and plant hormone signal transduction were prevalent and dominant. At the molecular level, the GO categories “metabolic process,” “cellular process,” “catalytic activity,” “binding,” “cell”and “cell part”, and the KEGG pathway categories “plant‒pathogen interaction”, “phenylpropanoid biosynthesis” and “MAPK signalling pathway” were significantly enriched under *C. cassiicola* stress (Fig. 8), which was consistent with findings in *Arabidopsis *[51], *Althaea officinalis*. L. [52], and rice [53].

Plant–pathogen interactions involve a series of defence measures to resist fungal invasion, such as allergic reactions, plant protection hormone synthesis and antibacterial protein production. In the formation of these defence mechanisms, the fastest resistance response after fungal recognition is an ROS burst, which produces a large number of ROS in a short time and plays a special role in the resistance response. In the interaction between wheat and stripe rust, ROS were only produced in resistant varieties. Under the stress of *C. cassiicola*, plants produce TFs and PKs to perceive stress signals, thus increasing the ROS concentration and activating ROS defence/metabolic pathways for ROS clearance. When plants were infected by *C. cassiicola*, KEGG pathway analysis showed that pathways involving peroxisome and glutathione metabolism, as well as hormone signal transduction, were all enriched. There is strong evidence that glutathione transferase is involved in the regulation of plant stress resistance. For example, GSTFs have been observed in *Arabidopsis *[54].

The aims of this study were to gain insights into the differences in the molecular mechanisms between resistant and susceptible varieties to cope with *C. cassiicola* infection and to identify potentially excellent disease-resistance genes in the resistant variety. It has been reported that starch and soluble sugars [55], phytohormones [56], and photosynthetic efficiency [57, 58] can be used as important reference indicators of plant phenotypic change. In this study, the changes of leaf spot expansion at different time points were recorded as phenotypic observation. If there are some physiological and biochemical indicators to be detected, it will be more helpful to improve the rigor of the experiment. In the observation of leaf phenotype, we found that mild spots were observed at 12 hpi in the S group, while group R did not develop symptoms until 48 hpi. By observing the changes of symptoms in leaves at 6–48 hpi, we selected key time points (12 h, 24 h, 36 h, and 48 h) to perform transcriptome sequencing by leaf tissue. Transcriptome data analysis found that a large number of genes were up-regulated at 12 hpi, indicating that 12 hpi was the key time point for *C. cassiicola* infection. Additionally, due to the advantages of WGNCA in gene function analysis, both differential expression analysis and WGNCA were used in the present study, as has been done for maize [59], tomato [60], wheat [61], and *Arabidopsis *[62]. In this study, a total of ten genes co-expression modules were identified by weighted gene co-expression network analysis, among which 2 (MEorangered4 and MEdarkmagenta modules) were specific modules related to resistance to *Corynespora* leaf spot. Then, we established resistance-regulated co-expression modules and identified some candidate transcription factors and protein kinases, such as WRKY, AP2/ERF-ERF, NAC, RLK-Pelle_DLSV, RLK-Pelle_SD-2b, and RLK-Pelle_WAK proteins, that may function in disease resistance.

TFs are proteins that can bind to specific nucleotide sequences upstream of a gene, which in turn can regulate the binding of RNA polymerase and DNA templates, thus regulating gene transcription. TFs are the master regulators of plant drought resistance. Many TFs, such as AP2/ERF, MYB, NAC, bHLH, C2H2-ZF, WRKY, and NF-Y TFs, have been confirmed to play roles in disease resistance in different plant species [63]. New members of some TF families, such as the C3H, G2-like, HSF, MYB-related, and MADS families, were found, except for some known TF families involved in plant disease-resistance [64–66]. Gao et al. [67] has reported that the transcription factor WRKY8 plays a positive regulatory role in plant immunity to pathogen infection and plant response to drought and salt stress. One study revealed that when plants perceive pathogen infection, they regulate the resistance mechanism of different plant protection factors against the invasion of pathogens through different signalling pathways [68]. On the one hand, the host plant activates the jasmonic acid and ethylene signalling pathways, regulating the synthesis of the plant protection factors scopoletin and scopolin. On the other hand, another important plant protection hormone, capsidiol, is regulated by the transcription factor ERF2-like. These results indicated that these TFs have a conserved function in different plant species to resist pathogen infection. In our study, TFs are key components in the plant signal pathway, and they are involved in the signal perception and the expression of downstream key genes in response to *C. cassiicola*. The transcriptome of sesame revealed that a number of different transcription factor families were affected by *C. cassiicola* stress, including members of WRKY, AP2/ERF, NAC, bHLH, MYB and so on. Our RNA-seq data showed that pathogen infection causes dramatic changes in gene expression profiles, and indeed, many plant defence hormone signals displayed differential expression within 12 hpi. In particular, AP2/ERF-ERF gene (LOC105176475, LOC105163850) expression was significantly increased in both resistant and susceptible varieties at 12 hpi. Similar transcription factors include NAC (LOC105159455, LOC105159980, LOC105167649, LOC105175173) and WRKY (LOC105167193, LOC105175676, LOC105176853, LOC105177346). These results indicate that AP2/ERF-ERF, NAC and WRKY TFs are involved in sesame disease resistance under *C. cassiicola* stress. In addition, the protein kinase gene (RLK-Pelle_DLSV) also showed the same tendency of high expression under *C. cassiicola* stress, and the gene expression amount in R was much higher than that in S. Therefore, it is speculated that these genes may constitute the key to the difference in disease resistance between resistant and susceptible varieties, which deserves more attention. Our analysis revealed a complex relationship between different time points after infection and the transcriptional responses of other resistance-related genes, implying that PK and TF can influence both of different sesame varieties. Our study provided valuable and nearly complete sequence information on sesame. Furthermore, we constructed a global molecular mechanism model of the resistance response in sesame (Fig. 11) with all this transcriptome information.

## Conclusions

In this report, we investigated the resistant or susceptible varieties and infection stage-specific response of sesame against *C. cassiicola* infection and found that 12 hpi was the key time point leading to the resistance difference between the susceptibility and resistance varieties. We also constructed gene co-expression networks for R and S by WGCNA and discovered that the MAPK signalling, plant–pathogen interaction, and plant hormone signalling pathway activity greatly influenced host resistance, and 10 candidate genes (Table S6) that potentially regulate sesame resistance and defence mechanisms were identified. This study provide a fine assembly of transcriptome data, which will pave the way for future research into sesame function at the molecular level and provide a rich resource for full-length genes that may be important for efforts aimed at improving the resistance of sesame.

## Methods

### Plant materials and inoculation of *C. cassiicola*

The sesame varieties used in the present research were Henan No.1 (resistant group, R) and Jinzhi No.3 (susceptible group, S), which were obtained from the Henan Sesame Research Center, Henan Academy of Agricultural Sciences, and the Institute of Economic Crops, Shanxi Academy of Agricultural Sciences, respectively. The sesame seeds were germinated in plastic pots (10 cm diameter, 9 cm height) containing a 3:1:1 mixture of field soil, peat soil, and vermiculite and then grown in a growth chamber (28–30℃) under a 16 h light/8 h darkness photoperiod. Repeat 3 pots at each inoculation time point and plant 5 seedlings in each pot. All the seedlings were inoculated after 2 months of growth.

C. *cassiicola* (Number: 20180909-03) was isolated and stored at the Institute of Plant Protection, Henan Academy of Agricultural Sciences. All the plants were surface inoculated with a spore suspension (10^6^ conidia/mL) applied by a hand-held spray bottle, and the inoculated plants were sealed with transparent plastic bags for 48 h. Leaves before inoculation (0 h) and leaves after inoculation (6 h, 12 h, 24 h, 36 h, 48 h) were taken to obtain sequencing samples. Samples for each group were harvested separately, immediately frozen in liquid nitrogen and stored at -80 °C until RNA extraction for transcriptome sequencing. The R group and S group contained 18 samples: six time points × three replicates each.

### Illumina transcriptome library preparation and sequencing

Total RNA was extracted using an RNeasy Mini Kit (Cat#74106, QIAGEN) following the manufacturer’s instructions, and the RIN was checked by an Agilent Bioanalyzer 2100 (Agilent Technologies, Santa Clara, CA, US). The qualified total RNA was further purified by an RNA Clean XP Kit (Cat A63987, Beckman Coulter, Inc., Kraemer Boulevard Brea, CA, USA) and an RNase-Free DNase Set (Cat#79254, QIAGEN, GmbH, Germany). The samples were sent to Shanghai Biotechnology Corporation for transcriptome sequencing. The samples were also used for qRT‒PCR analysis.

The starting sample of the sequencing experiment was total RNA, which was inspected via a NanoDrop ND-2000 spectrophotometer and Agilent Bioanalyzer 2100 (Agilent Technologies, Santa Clara, CA, US). The qualified RNA was used for library construction. Library construction of sequencing samples involved mRNA separation, fragmentation, first-strand cDNA synthesis, second-strand cDNA synthesis, end repair and other steps involving the purified total RNA.

For all the constructed libraries, a Qubit^®^ 2.0 Fluorometer was used to measure concentration, and an Agilent 4200 was used to measure library size. The qualified library was then sequenced. Sequencing reagents were prepared according to the Illumina User Guide, and the cluster flow cell was loaded onto the machine. The paired-end program was used for paired-end sequencing. The sequencing process was run by Illumina data collection software, and real-time data analysis was performed.

### Illumina data analysis

The low-quality reads were filtered with Seqtk (https://github.com/lh3/seqtk) by removing the adapter sequence, and the obtained high-quality clean reads were used for genome alignment. The spliced mapping algorithm of HISAT2 (version: 2.0.4) [69] was applied to conduct genome mapping for the preprocessed reads. Then, To achieve comparability of gene expression levels between different genes and samples, reads were transformed into (fragments per kilobase of exon model per million mapped reads) FPKM for standardization of gene expression levels. The reads mapped to gene regions were converted into FPKM for standardization of gene expression [70]. edgeR [71] was applied to conduct differential gene analysis among samples, and genes identifed by edgeR with FDR ≤ 0.05 and FC ≥ 2 were defned as diferentially expressed.

### PacBio SMRTbell library preparation and sequencing

The high-quality RNA was reverse transcribed into cDNA with a SMARTer^®^ PCR cDNA Synthesis Kit, and PCR amplification was performed using KAPA HiFi PCR Kits. The PacBio Biosciences SMRT-seq platform provides long reads up to transcript length. A SMRTbell library was constructed with a SMRTbell Template Prep Kit(1.0). The DNA was repaired by DNA Damage Repair Mix (PacBio) and End Repair Mix (PacBio). Stem loop sequencing splices were attached to both ends of the cDNA fragments, and exonuclease was used to remove the fragments that failed to connect. After library construction, the library templates and enzyme complexes with certain concentrations and volumes were transferred to the nanopore of the PacBio Sequel sequencer for single molecule real-time (SMRT) sequencing. After sequencing was completed, high-quality sequencing data were filtered, and a series of biological information analyses was carried out. SMRTlink (version 6.0) analysis software [72] was used to process the original data, and full-length consensus sequences were obtained after clustering. Then, the full-length consensus sequences were used for subsequent analysis.

### PacBio data analysis

The analysis was based on the IsoSeq3 (https://github.com/PacificBiosciences/IsoSeq3) process. The raw polymerase reads that had a minimum number of full passes ≥ 1 and a minimum prediction accuracy ≥ 0.80 were selected for producing reads of insert (ROIs). Among the sequencing results, not all ROIs were complete, and a small part of the sequences were chimaeras. After obtaining the full-length transcript sequence, further processing was needed to remove the terminal poly-A and chimaeras to obtain full-length nonconcatemer reads.

### Functional annotation of transcripts

DIAMOND software (version 2.0.4, parameter: -k 100 -e -evalue 1e-5 -f 5) was used to compare the obtained new transcript sequences with those in the NR (ftp://ftp.ncbi.nih.gov/blast/db/), SwissProt (http://www.uniprot.org/), GO (http://www.geneontology.org/), COG (http://www.ncbi.nlm.nih.gov/COG/), KOG (http://www.ncbi.nlm.nih.gov/COG/), Pfam (http://pfam.xfam.org/) and KEGG (http://www.genome.jp/kegg/) databases to obtain annotation information of the transcripts.

### Identifcation of AS events, lncRNAs and SSR analysis

We used Astalavista software (http://astalavista.sammeth.net/) [73] to obtain the variable splicing types existing in each sample.

Four computational methods, namely, those associated with the Pfam, Cooperative Data Classification (CPC) [74], Coding Assessing Potential Tool (CPAT) [75], and Coding Non-Coding Index (CNCI) [76] databases, were used to identify long non-coding RNAs (lncRNAs). MISA (MIcroSAtellite identification tool) is a software that identifies simple repeat sequences (http://pgrc.ipk-gatersleben.de/misa/), it can identify seven types of SSR by analyzing transcript sequences.Transcripts longer than 500 bp were screened from the new transcripts, and SSR analysis was performed using MISA (version 1.0, Parameter: Default).

### Differentially expressed gene identification

To identify differentially expressed genes (DEGs), we used DESeq (http://www.bioconductor.org/packages/release/bioc/html/DESeq.html) to analyse gene expression differences between the R and S group. A fold = change ≥ 2 and an FDR < 0.01 were used as screening criteria in the detection of differentially expressed genes.

### WGCNA and Cytoscape analysis

The WGCNA package (https://horvath.genetics.ucla.edu/html/CoexpressionNetwork/Rpackages/WGCNA/index.html) was used to construct a co-expression network for transcriptome analysis. Then Pearson correlation matrix and network topology analysis were used to calculate the gene correlation of the samples at 6 time points (0, 6, 12, 24, 36, 48 h). Use the following Settings: minimum module size of 50, absolute value of correlation coefficient ≥ 0.3 and the threshold value of *p*Value < 0.05, the modules related to each trait were screened. For each trait related module, the correlation of module Gene expression and corresponding trait (Gene Significance, GS) was calculated respectively, and the correlation between module gene expression and Eigengene was also calculated.

By using Cytoscape (version 3.7.1) and plugin of Cyto-Hubba, the central and highly connected genes of specific modules in each stage were identified by visualizing the top 20 genes. Three computational algorithms of Cyto-Hubba named Degree, Closeness, and MCC were used for detecting hub genes.

### Quantitative real-time PCR

The primers used for quantitative real-time PCR (qRT‒PCR) were designed by Primer Premier 5.0 software [77] and synthesized by Sangon Biotech; they are shown in Table S7. A CFX 384™ real-time system (Thermo) was used to measure fluorescence quantities with a reaction system of 10.0 μL comprising 5.0 μL Luna^®^Universal qPCR Master Mix, 0.25 μL of forward and reverse primers, 1.0 μL of cDNA and 3.5 μL of RNase-Free dH_2_O. PCR amplifcation was carried out as follows: First, 95 °C for 3 min. Secondly, followed by 40 cycles of 95 °C for 10 s, 58 °C for 30 s, 72 °C for 30 s. Finally, 65 °C for 5 s, 95 °C for 5 s. Relative quantitation was calculated with the 2^−ΔΔCt^ method [78]. Each sample was replicated 3 times.

### Statistical analysis

All the data in this study are the mean values of three biological replicates. The expression level of each gene was normalized to fragments per kilobase per million (FPKM) for comparison between different samples. The statistical analysis of the qRT‒PCR data in this study was performed using SPSS 13 software [79], and the mappings were generated using Excel 2010.

### Supplementary Information

**Additional file 1: Table S1.** CCS sequence was extracted from the original sequence.

**Additional file 2: Table S2.** Full-length sequence data statistics table.

**Additional file 3: Table S3.** Consensus isoforms data statistics table.

**Additional file 4: Table S4.** Summary of annotated transcripts.

**Additional file 5: Table S5.** Statistics of differentially expressed gene.

**Additional file 6: Figure S1.** PCA diagram of 36 samples.

**Additional file 7: Figure S2.** TopGO analysis between R and S at 12 hpi.

**Additional file 8: Table S6.** The expression and annotation of candidate genes.

**Additional file 9: Table S7.** The primers used for qRT‒PCR.

## Data Availability

The sequenced raw reads generated in this study have been submitted to the National Center for Biotechnology Information (NCBI) with BioProject ID: PRJNA961921 (https://dataview.ncbi.nlm.nih.gov/object/PRJNA961921?reviewer=g3atcgj1cpce1tano8e6tmbemm). Additional analytical data during this study are included in the supplemental information.

## Abbreviations

*C. cassiicola*: *Corynespora cassiicola*
CLS: *Corynespora* leaf spot
hpi: Hours post inoculation
AS: Alternative splicing
DEGs: Differentially expressed genes
FPKM: Fragments Per Kilobase Million
NR: Non-redundant protein sequence database
Swiss-Prot: A manually annotated, non-redundant protein sequence database
KOG: The database of Clusters of Protein homology
COG: The database of Clusters of Orthologous Groups of proteins
GO: Gene Ontology
Pfam: Protein families database
KEGG: The database of Kyoto Encyclopedia of Genes and Genomes
WGCNA: Weighted Gene Co-Expression Network Analysis
TF: Transcription Factor
PK: Protein kinase
qRT-PCR: Quantitative real time polymerase chain reaction
