# Supplementary figures and images for: Full-length transcriptome and RNA-Seq analyses reveal the resistance mechanism of sesame in response to Corynespora cassiicola
Source: BMC Plant Biol. 2024 Jan 23;24:64. doi: 10.1186/s12870-024-04728-y (PMC10804834; doi:10.1186/s12870-024-04728-y)

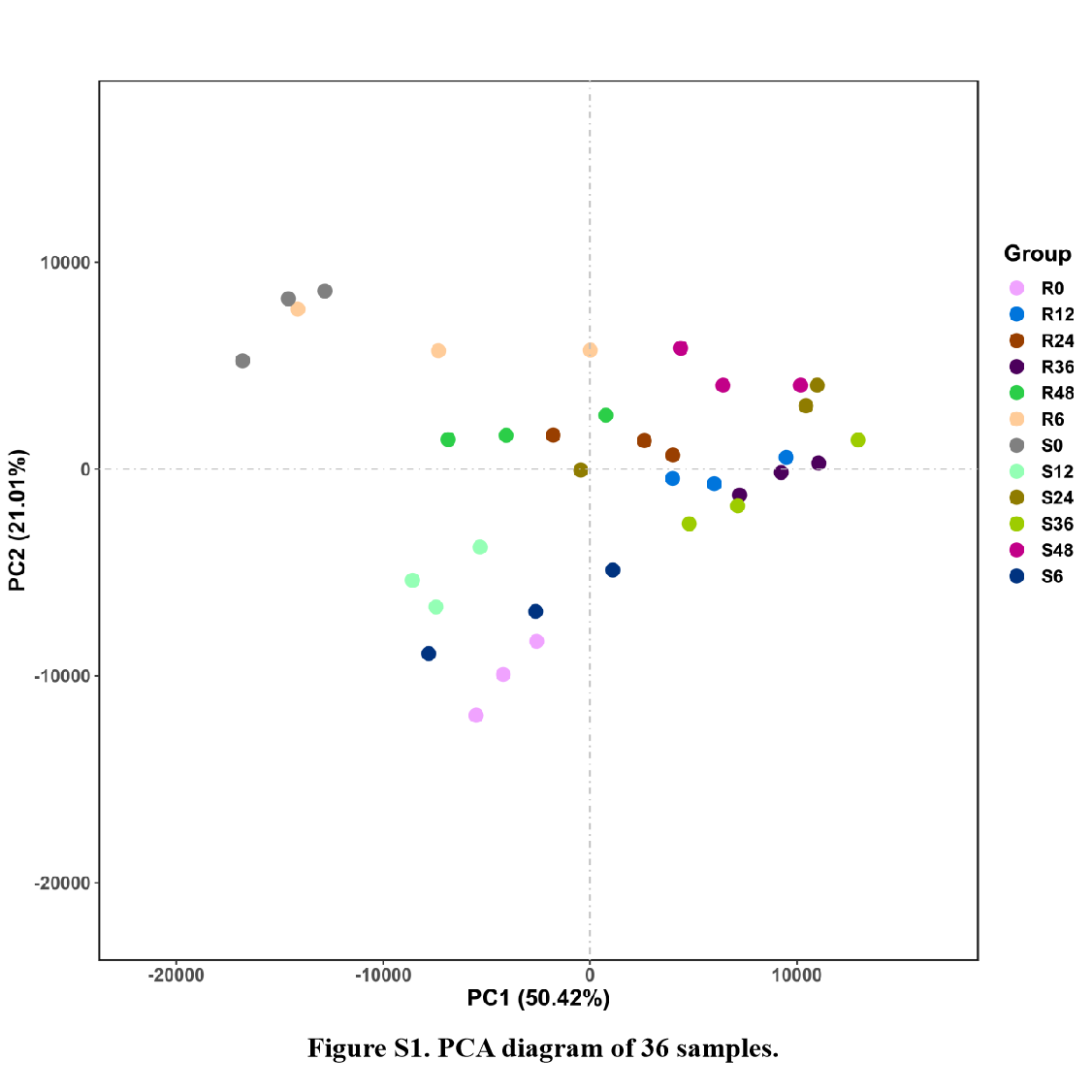

Supplement: Supplementary file 6 — Additional file 6: Figure S1. PCA diagram of 36 samples. [file 12870_2024_4728_MOESM6_ESM.tif]

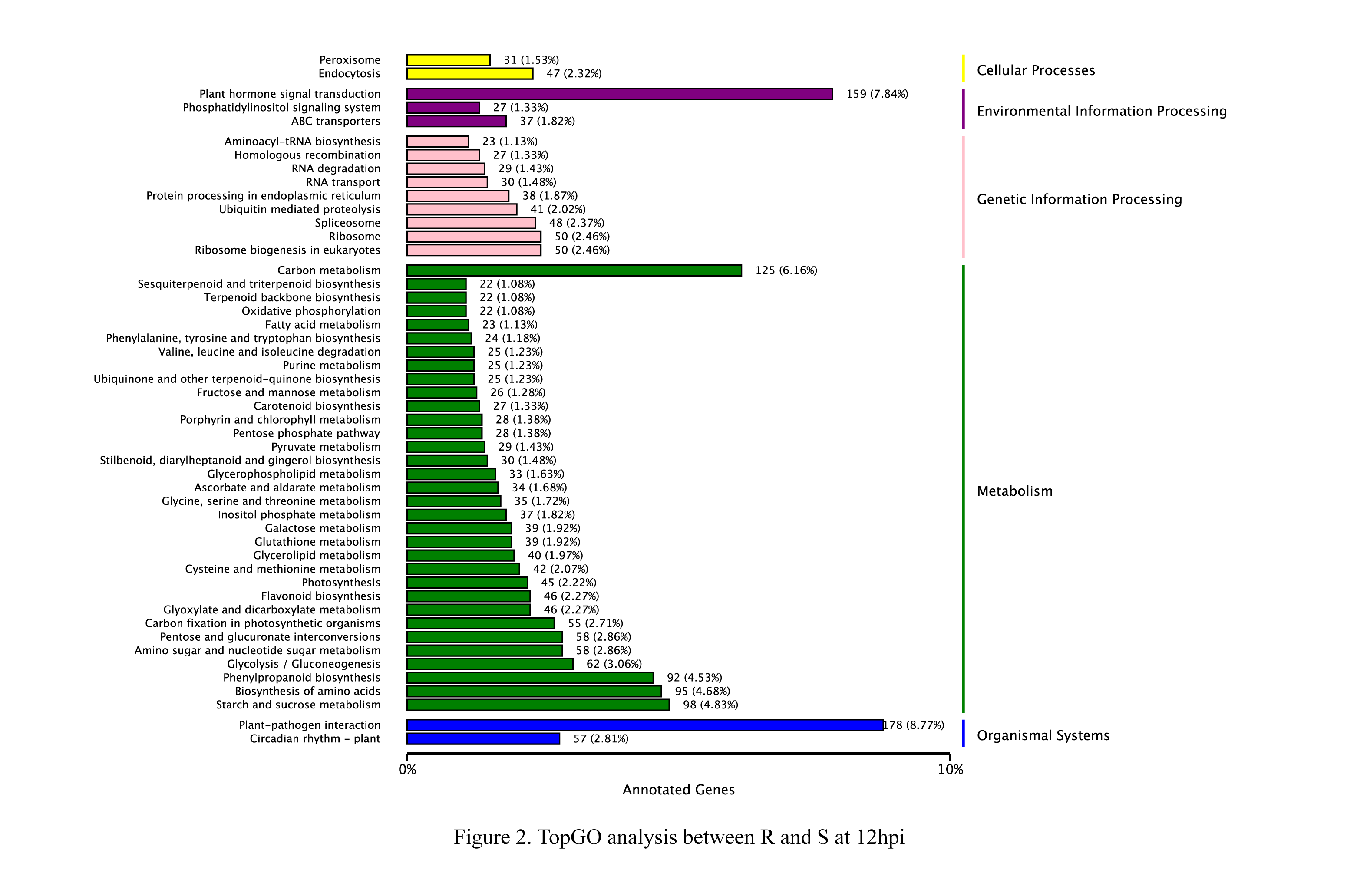

Supplement: Supplementary file 7 — Additional file 7: Figure S2. TopGO analysis between R and S at 12 hpi. [file 12870_2024_4728_MOESM7_ESM.tif]
